# 2-Phenylcyclopropylmethylamine (PCPMA) Derivatives as D_3_R-Selective Ligands for 3D-QSAR, Docking and Molecular Dynamics Simulation Studies

**DOI:** 10.3390/ijms26083559

**Published:** 2025-04-10

**Authors:** Li Guo, Yuepeng Gao, Sujuan Zhang, Lingmi Zhao, Runxin Zhao, Pinghua Sun, Xinhui Pan, Wei Zhang

**Affiliations:** 1 Key Laboratory of Xinjiang Phytomedicine Resource and Utilization Ministry of Education, School of Pharmacy, Institute for Safflower Industry Research, Shihezi University, Shihezi 832002, China; gl20180108@163.com (L.G.); gyp2244371141@outlook.com (Y.G.); z987654321sj@163.com (S.Z.); 15199595015@163.com (L.Z.); 19301306330@163.com (R.Z.); pinghuasunny@163.com (P.S.); 2 International Cooperative Laboratory of Traditional Chinese Medicine Modernization, Innovative Drug Development of Chinese Ministry of Education (MOE), College of Pharmacy, Jinan University, Guangzhou 510632, China; 3 Stake Key Laboratory of Natural and Biomimetic Drugs, Department of Chemical Biology, School of Pharmaceutical Sciences, Peking University, Beijing 100191, China

**Keywords:** D_3_R, PCPMAs, 3D-QSAR, ADMET, molecular docking, molecular dynamics simulation

## Abstract

Dopamine D_3_ receptor (D_3_R) is a key receptor for regulating motor, cognitive, and other functions. In this study, 50 2-phenylcyclopropylmethylamine (PCPMA) derivatives with good selectivity for D_3_R were investigated using a three-dimensional quantitative structure–activity relationship (3D-QSAR) method. The CoMFA and CoMSIA model results showed good predictive ability, as evidenced by high r^2^ and q^2^ values. 3D-QSAR results showed that steric, electrostatic, and hydrophobic fields played important roles in the binding of PCPMAs to D_3_R. Based on above results, four novel PCPMAs were designed, which were predicted to have a stronger affinity with D_3_R. Molecular docking combined with 300 ns molecular dynamics simulations were performed to reveal the mode of interaction between D_3_R and PCPMAs. Additionally, a combination of free energy calculations and energy decomposition results indicated strong interaction between the ligands and residues in the binding pocket of the D_3_ receptor. This work provides suggestions for exploring more selective D_3_R ligands, and this theoretical framework also lays the foundation for future experimental investigations to evaluate the pharmacological characteristics and binding affinities of novel derivatives.

## 1. Introduction

Dopamine (DA) receptors are widely distributed in the brain and are one of the most important neurotransmitters in the nervous system. These receptors are G protein-coupled receptors (GPCRs) that play vital roles in various functions such as motor control, motivation, and cognitive processes. They have been classified into two subfamilies, D_1_-like (D_1_ and D_5_ receptors) and D_2_-like (D_2_, D_3_, and D_4_ receptor subtypes), on the basis of sequence differences, activation of G-protein-coupled receptors, and pharmacological properties. The D_1_-like receptors activate a stimulatory G protein-coupled receptor to increase adenylate cyclase activity, while D_2_-like receptors activate inhibitory G protein alpha subunit (G_i/o_)-coupled receptors to inhibit adenylate cyclase and modulate other effector pathways [1,2,3]. D_2_ and D_3_ receptors (D_2_R and D_3_R), belonging to the D_2_-like category, are particularly important as therapeutic targets for neuropsychological disorders like schizophrenia, Parkinson’s disease, and drug addiction. Because of the high degree of sequence identity (78%) within the transmembrane (TM) domains and the close identity of the residues that form the binding site with the receptor, this makes it challenging to create subtype-selective agents with physicochemical properties suitable for in vivo characterization of the physiological actions of these receptor subtypes [4]. D_2_R is the primary target for traditional antipsychotic medications, but the presence of antagonism with the cerebral striatum has led to the emergence of side effects, including extrapyramidal symptoms (EPS) [3], elevated prolactin, drug abuse, and damaging effects on motivated behavior. D_3_R has been a therapeutic target of great interest due to its relatively localized expression in the mid-limbic neural circuits, including the nucleus accumbens, hippocampal island of Calleja, ventral striatum, and the selective distribution of D_3_R in the nucleus ambiguus. In particular, it has the potential to reduce the incidence of side effects such as EPS and hyperprolactinemia, which has enhanced the interest in D_3_R as a potential target for drug addiction [5]. As a result, there is a pressing need for the design and development of highly selective D_3_R ligands to address psychiatric disorders [6,7,8].

**Figure 1:**
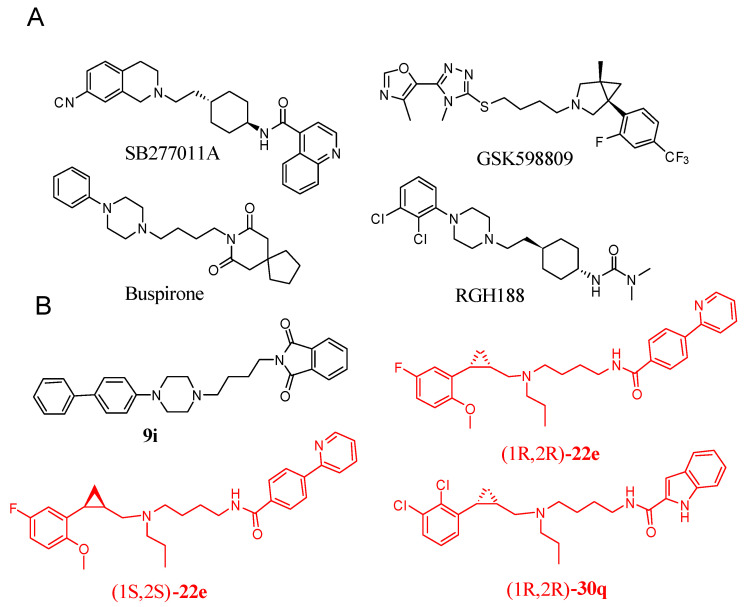
(**A**) D_3_R-selective ligands in clinic and clinical trials. (**B**) The bitopic ligands of D_3_R.

D_3_R-selective ligands in clinic and clinical trials are shown in Figure 1A. SB277011A, introduced in 2000 [9], has become an essential scaffold in the study of dopamine D_3_ receptors (D_3_R). As a competitive D_3_R-selective antagonist, it had been employed extensively in vivo to elucidate the role of D_3_R in various neural circuits and its potential for therapeutic applications in neuropsychiatric disorders. In 2010, Micheli et al. identified GSK598809 as a D_3_R antagonist, notable for its exceptional 100-fold selectivity for D_3_R over D_2_R. This compound belonged to a new 1,2,4-triazol-3-yl-azabicyclo[3.1.0]hexanes backbone with a favorable pharmacokinetic profile, but a potential cardiovascular risk was associated with high doses of GSK598809 in clinical trials [10]. Buspirone, a D_3_R-preferring 5-HT 1A antagonist, but it was noteworthy that its selectivity for D_3_R was only observed with oral administration, suggesting a relationship with D_3_R that could be attributed to the metabolism of buspirone. It appeared that the active fraction contributing to its D_3_R selectivity may be a metabolite generated during its first pass through the liver [11]. RGH188 (cariprazine, CRP) was also a D_3_R-preferring agent, showing a 10-fold selectivity for D_3_R over D_2_R. Noteworthily, cariprazine was shown to have cognitive enhancement in vivo, further underscoring the therapeutic potential of D_3_R-selective ligands in treating cognitive deficits common in neuropsychiatric disorders [12].

Despite progress in developing highly selective D_3_R ligands, challenges such as low bioavailability, poor brain permeability, and adverse side effects observed in clinical trials remain [8,10]. These issues emphasize the necessity for continued research targeting D_3_R for drug development, as effective and safe therapeutic options are still needed for neuropsychiatric disorders. Currently, the design of D_3_R-selective ligands is mostly based on the strategy of using the unique characteristics of the D_3_R binding pocket to create new compounds that can engage in multi-faceted interactions, potentially leading to enhanced selectivity and affinity [10,13,14]. Inspired by fragmentation and molecular docking, Cheon et al. designed and synthesized a series of piperazine–phthalimide bitopic ligands, and found that compound **9i** exhibited a high D_3_R selectivity (100-fold higher than D_2_R) and 199-fold increase in agonist activity compared to quinpyrazole [15]. Shaik et al. employed the crystal structure of the D_3_R complexed with eticlopride to design a novel bitopic ligand, and highlighted that the pyrrolidine ring O-alkylated derivatives showed high affinity with D_2_R/D_3_R [16]. Francisco O Battiti et al. found that FOB02-04A is a bitopic and full agonist of D_3_R. Meanwhile, a selectivity site was revealed by determining the cryo-electron microscopy (cryo-EM) structure of the hD_3_R:Gα_O_βγ complex bound to the D_3_R selective bitopic agonist FOB02-04A [17]. Besides, PCPMA derivatives have been reported as serotonin transporter inhibitors [18], melatonin receptor agonists [19], 5-HT2C receptor agonists [20], etc, which have potential value in the treatment of neurological disorders such as schizophrenia and addiction [21,22]. Recently, a series of PCPMAs, particularly compounds (1*R*,2*R*)-**22e** and its enantiomer (1*S*,2*S*)-**22e**, were designed as selective D_3_R ligands. Among them, compound (1*R*,2*R*)-**30q** showed a significant affinity for D_3_R with a *K*_i_ value of 2.2 nM in mice. These compounds were strategically designed by incorporating a linker that enhanced binding to D_3_R and a moiety that bound to the extended binding pocket (EBP) of the receptor [23].

As a 5-HT 2C receptor agonist, a PCPMA derivative modified by the virtual screening lead compound cyclopropylamine showed antidepressant-like effects in commonly used behavioral assays, similar to the potential therapeutic effect of D_3_R [20]. The clinical extended-release melatonin preparation tasimelteon containing a PCPMA backbone also had similar effects to D_3_R in neuroprotection and drug abuse by activating two high-affinity G protein-coupled receptors, Melatonin Receptor 1 (MT1) and Melatonin Receptor 2 (MT2) [19]. This highly selective PCPMA 5-HT 2C agonist compound also showed moderate binding affinity for dopamine D_3_R. Therefore, Chen et al. linked the junction moiety that binds well to D_3_R and the group that binds to the extended binding pocket (EBP) to the nitrogen atom of PCPMA, and the designed compounds showed high binding affinity for D_3_R, good selectivity, bioavailability, and brain penetration. Therefore, we selected these compounds for research in order to obtain D_3_R ligands with better selectivity and higher affinity [23].

3D quantitative structure–activity relationship (3D-QSAR) studies have been found to be of great significance in ligand-based drug design and potent drug development, which correlates 3D-structural features of the chemicals with their associated properties [24,25,26]. Comparative molecular field analysis (CoMFA) [27] and comparative molecular similarity index analysis (CoMSIA) [28] have been employed. The former correlated steric and electrostatic properties of the molecular structure with its activity, while the latter correlated the hydrophobic field, hydrogen-bond donor/hydrogen-bond acceptor and steric/electrostatic field with its activity.

**Scheme 1:**
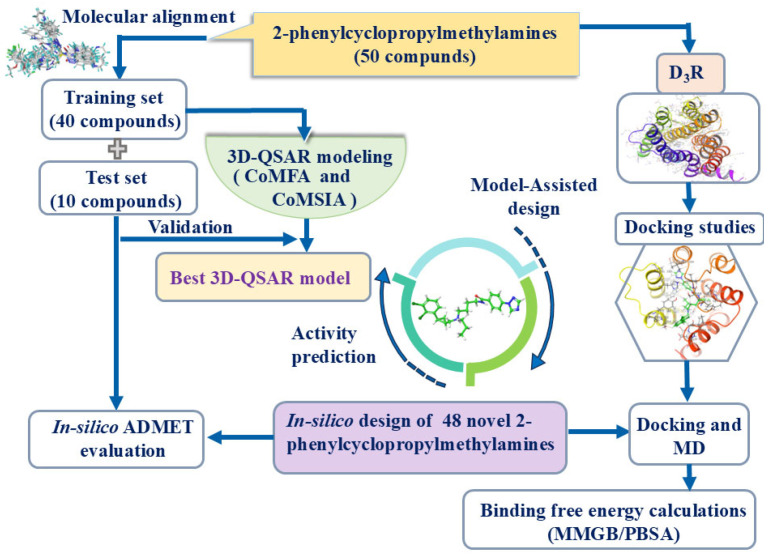
The workflow of the current study.

Given the potential of PCPMAs as D_3_R ligands, an in-depth computational study of a series of PCPMAs was performed to explore their quantitative structure activity relationship and further improve their selectivity and affinity for D_3_R. To establish a robust dataset, the affinities (p*K*_i_) and molecular structures of 50 compounds reported by Cheng’s group were selected [23] (see Table S1). Based on the results of the 3D-QSAR modeling and contour map analysis, a series of novel derivatives were designed, and their binding affinities with D_3_R were predicted. The outcomes of this work were expected to enhance the understanding of the structure–activity relationships of PCPMA derivatives and provide a reliable framework for predicting the biological activity of new candidates. Taken together, our results contribute to the design of novel D_3_R ligands and provide a theoretical basis for the discovery of molecules with potential antipsychotic activity. The workflow of the current study is represented in Scheme 1.

## 2. Results and Discussion

### 2.1. CoMFA and CoMSIA Statistical Results

**Figure 2:**
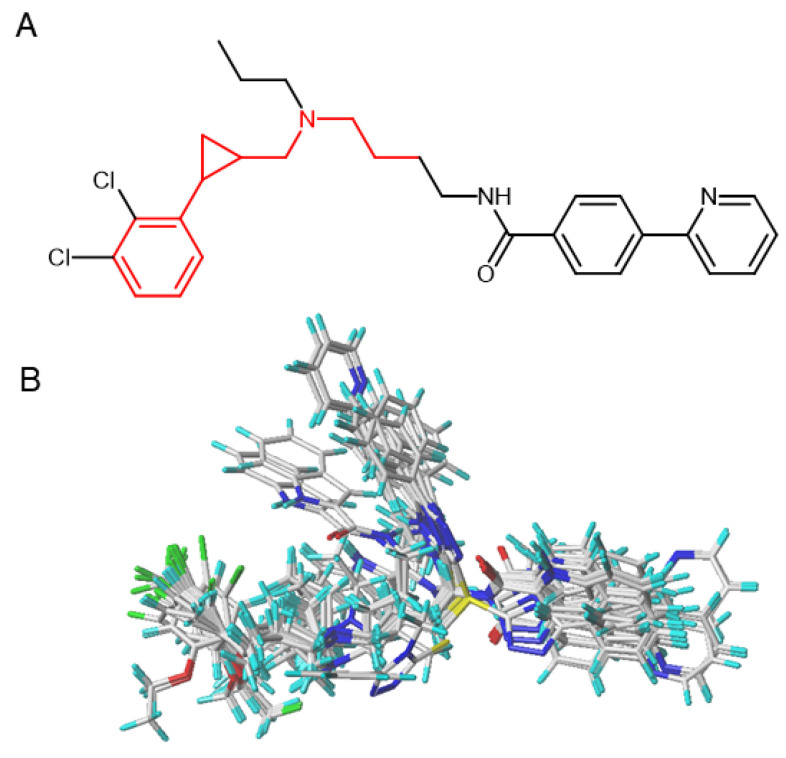
(**A**) The structure of compound **30r** and structure-based aligned common substructures are shown in red bold lines. (**B**) Structural superposition of 50 ligands for 3D-QSAR modeling.

A 3D-QSAR study was performed on 50 PCPMA derivatives in this work. All molecules were listed in Table S1. The compounds in the training set were aligned to compound **30r**, which had the highest affinity. It could be seen from Figure 2 that the common skeleton of all molecules overlapped. One CoMFA model and 32 CoMSIA models were developed by the 3D-QSAR study. The statistical parameters of CoMFA and the best CoMSIA models are listed in Table 1. The best CoMSIA model, combining steric and hydrophobic fields, and the CoMFA model were selected to predict the binding affinity of compounds with D_3_R. The predicted affinity (p*K*_i_) values of the training and test sets are shown in Table S1.

To give the 3D-QSAR model a stable and reliable prediction ability, the q^2^ of the constructed model was generally larger than 0.5, while r^2^ was close to 1. The smaller the SEE value, the better the quality. For the CoMFA model, q^2^ was 0.607, N was 10, r^2^ was 0.981, SEE was 0.094, and F was 149.222, respectively. In summary, the CoMFA model in this study was reliable, and the contributions of the steric and electrostatic fields were 39.3% and 60.7%, respectively, indicating that the electrostatic field had an important effect on the binding affinity of the PCPMA ligands with D_3_R. This may be consistent with the two-step mechanism of GPCR ligand binding—electrostatic interactions determine binding specificity (orientation), while hydrophobic interactions provide binding energy (stabilization) [29,30].

For the CoMSIA model, 32 models with different field combinations were obtained (see Table S2). The combination of electrostatic and steric fields was selected as the best CoMSIA model due to its q^2^ of 0.643, r^2^ of 0.889, and the presence of four components. These statistics showed that the developed CoMSIA model has good reliability. In this model, the contributions of the steric and hydrophobic fields on compound affinity were 37.5% and 72.5%, respectively, indicating that hydrophobic interactions had an important role in the binding affinity of PCPMA ligands with D_3_R. It is generally known that CoMSIA models tend to outperform CoMFA models, but in our study, CoMFA performed better in this case. The nature of the molecules in our dataset might be more suitable for the CoMFA approach. CoMFA focuses on steric and electrostatic fields, which could capture the key interactions relevant to our set of compounds more effectively than the CoMSIA model, perhaps because the steric and electrostatic effects dominate the binding mechanisms of our compounds. And our dataset is relatively small, so the CoMFA model might be more robust and less prone to overfitting compared to the more complex CoMSIA model [31,32,33].

**Figure 3:**
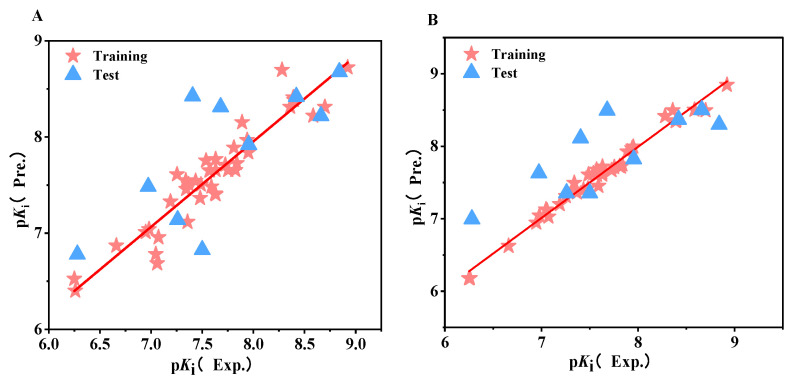
Scatter plots of experimental and predicted affinity (p*K*_i_) for (**A**) CoMFA and (**B**) COMSIA models.

The predicted values of p*K*_i_ for all compounds in the test set, calculated using CoMFA and the best CoMSIA model, are shown in Table S1, and the difference between the experimental and predicted affinity was found to be small. The correlation between the predicted affinity of the models and the experimental affinity of the training and test sets was then verified using scatter plots of the regression curves. As shown in Figure 3, most of the points were evenly distributed on both sides of the regression curve, which further proves the reliability of the 3D-QSAR model.

### 2.2. CoMFA and CoMSIA Contour Map Analysis

**Figure 4:**
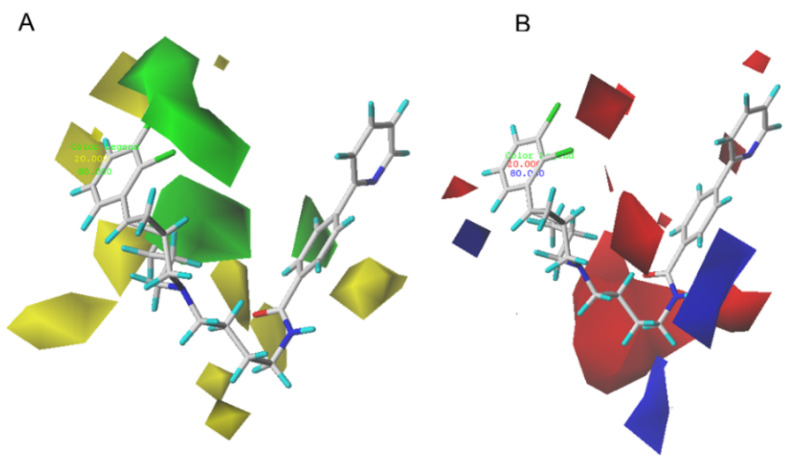
(**A**) CoMFA steric field and (**B**) electrostatic field contour map of **30r**.

**Figure 5:**
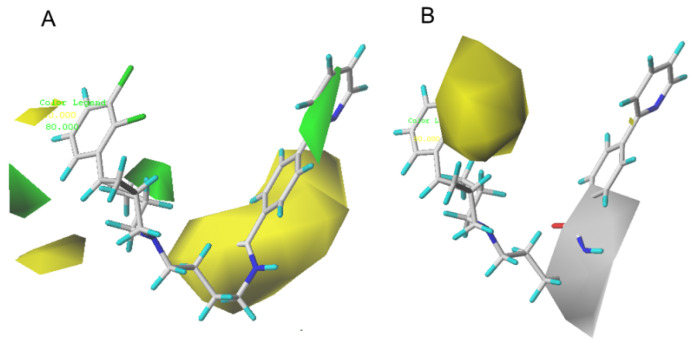
(**A**) CoMSIA steric field and (**B**) hydrophobic field contour map of **30r**.

To investigate the structure–activity relationship between various factors and compound structures in 3D-QSAR modeling, contour maps of CoMFA and optimal CoMSIA models were constructed based on template compound 30r (See Figure 4 and Figure 5). The contributions of favorable and unfavorable zones in all contour maps were 80% and 20%, respectively.

The CoMFA modeling results show that the binding affinity of PCPMA ligands with D_3_R was related to the spatial and electrostatic interactions of the ligands. The contour plots of the steric field are shown in Figure 4A, where green and yellow contours indicate that bulky groups were favorable and unfavorable for affinity, respectively. There was a large green contour near the phenyl and in the methylamine side chain, suggesting that the presence of bulky groups favored the activity. The contour plot of the electrostatic field is shown in Figure 4B, where the blue and red contours display whether positive charge was favorable or unfavorable for activity, respectively. There is a red contour at the end, attached to the amide bond of the methylamine linker chain, indicating that a negatively charged substitution could increase the affinity.

The best CoMSIA model results suggest that the magnitude of the affinity between the PCPMA ligands and the D_3_R was influenced by the molecular steric structure field and hydrophobic interactions. For the CoMSIA model, this mainly consists of a green region at the methylamine N side chain substitution, where the introduction of a bulky group may improve compound affinity, consistent with the CoMFA model; a large yellow region at the amide bond at the end of the fatty linkage chain and at the aryl substitution of the linked aryl group, suggesting that the affinity of the compound may be improved by the introduction of a smaller bulky group here; and finally a smaller green area at the aryl rear substituent, where the addition of a bulky group could be attempted to increase the affinity (shown in Figure 4A and Figure 5A). Furthermore, contour plots of the hydrophobic field are shown in Figure 5B, where yellow and grey contours indicate the favorable and unfavorable affinity of the hydrophobic groups, respectively. There is a large yellow contour at the vicinity of the phenyl group, indicating that the presence of the hydrophobic group was conducive to increasing affinity, and a large grey contour near the amide bond of the methylamine linkage chain, demonstrating that the presence of hydrophilic groups favored affinity.

### 2.3. Design of Novel PCPMA-Like Compounds

**Figure 6:**
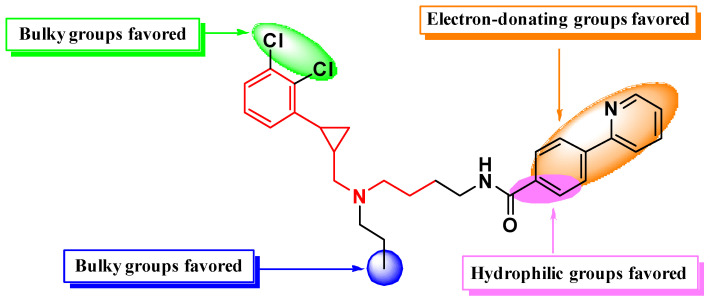
Structural modification sites of compound **30r**.

Based on the information obtained from the above contour plots, the key points of the subsequent structural modification of the compound **30r** with the best affinity are summarized (see Figure 6). Firstly, the substituents on the benzene ring of the cyclopropyl side chain of the PCPMA ligands preferred bulky hydrophobic groups such as halogenated hydrocarbons, aryl rings, and so on. Secondly, the affinity of the PCPMA ligands with D_3_R was enhanced when bulky groups such as benzene, biphenyls, and amino acids were present on the N-propyl side chain. Lastly, the affinity of the compounds could be increased when the group attached to the amide bond was a negatively charged hydrophilic group.

Based on the above results, a series of novel PCPMA ligands were designed, as shown in Table 2. The designed compounds **D1** to **D4** exhibited good predicted affinities compared to template compound **30r**, and more in-depth studies were conducted on these four compounds. The nitrogen-containing aliphatic linkage chain terminal amide bond connecting 1-phenyl-1-H-1,2,4-triazole and imidazole favored the compounds’ affinity (see compounds **D1**, **D4** in Table 2), and the affinity of the compounds designed by the addition of nitrogen atoms between the nitrogen-containing aliphatic linkage chain terminal amide bond and 1-phenyl-1-H-1,2,4-triazole also increased (see compound **D2** in Table 2). The activity of compounds with bulky groups such as biphenyl compounds was also increased by the addition of bulky groups to the side chains on the nitrogen atoms of the connecting chains (see compound **D3** in Table 2). Comparable binding affinities between the newly designed compounds **D1** to **D4** require further experimental validation.

### 2.4. ADMET and Drug Similarity Prediction Results

The ADMET performance of the newly designed compounds and the template compound were predicted using the ADMET lab 3.0 online website [34], and the drug properties and synthetic accessibility scores of the new compounds were also evaluated. As shown in Table 3, the LogP of all the designed compounds were smaller, and the topological polar surface area (TPSA) was used to assess the solubility of the compounds, the blood–brain barrier permeability, and the protein binding; all compounds scored within the normal range. The synthesis accessibility scores of the newly designed compounds were all less than 6, suggesting that all designed compounds could easily to be synthesized. As shown in Table S2, the oral bioavailability (F30%), as the crucial parameter of absorption for orally administered drugs, was 0–0.1, indicating excellent oral bioavailability. As shown in Table S3, the plasma protein binding (PPB) and volume of distribution (Vd) were calculated to describe the distribution behavior; all designed compounds exhibited PPB values > 90%, indicating high levels of plasma protein binding and probable low therapeutic indexes. Vd values are a parameter characterizing the duration of drug retention in plasma or redistribution into other tissues [35], which were in the range 0.04–20.00 L/kg in our study; all compounds exhibited Vd values in the range of 0.40–1.00 L/kg. Concerning metabolism, one of the most important metabolizing enzyme systems is cytochrome P450 (CYP) [36]. The results show that all compounds were substrates of CYP2C19, CYP2C9, and CYP2B6 isoforms, which may cause drug–drug interactions. Finally, regarding excretion, the half-lives (T_1/2_) of the compounds were used to assess their excretion rates. Drugs with T_1/2_ values close to 1 were characterized by a long half-life (>3 h), and T_1/2_ values close to 0 meant a shorter half-life (<3 h). The results reveal the tendency for half-life value reduction in the following order: **D4** > **D2** > **D1** > **D3**. The combined ADMET results show that compounds **D1**, **D2**, and **D4** fully comply with Lipinski’s [37], Ghose’s [38], Veber’s [39], Egan’s [40], and Muegge’s [41] rules, while compound **D3** exceeds the molecular weight limit due to its naphthalene extension. However, its other properties (e.g., low LogP, acceptable TPSA) suggest potential bioavailability.

### 2.5. Molecular Docking

**Figure 7:**
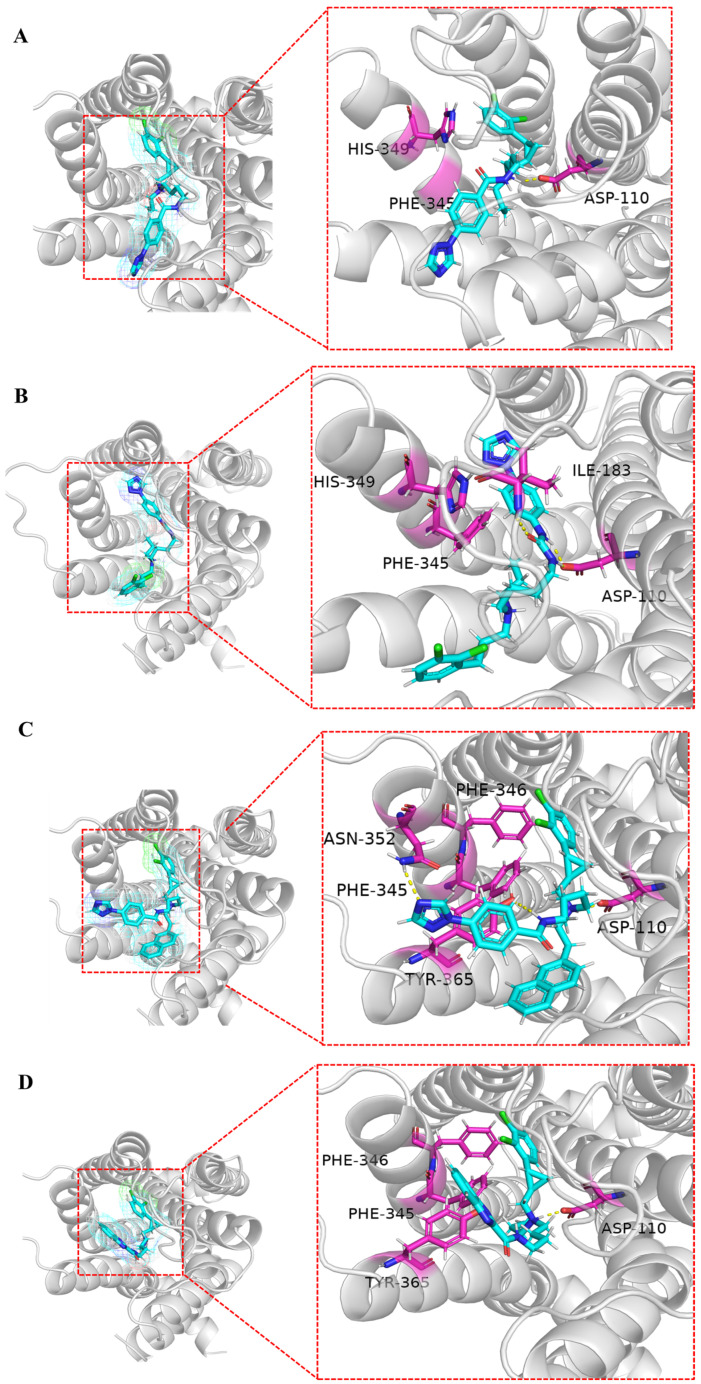
Predicted three-dimensional (3D) binding models of designed compounds (**A**) **D1**, (**B**) **D2**, (**C**) **D3**, and (**D**) **D4** with D_3_R.

In order to investigate the difference in binding affinity and binding modes in various ligand–D_3_R complexes, interaction analyses were performed for the molecular docking results of **D1**, **D2**, **D3**, and **D4** with D_3_R (see Figure 7). Compared with the template compound **30r**, all designed compounds were scored better for docking with D_3_R than D_2_R (see Table S4), except compound **D2**, indicating that all compounds could bind to D_3_R more stably and form stronger binding interactions.

As shown in Figure 7, the docking results demonstrate that all the designed compounds exhibited stable binding within the active pockets of D_3_R, establishing various interactions with key residues. Notably, the left PCPMA motif of compounds **D1**, **D3**, and **D4** was located at the orthosteric binding sites (OBS), which were characterized by hydrophobic pockets and surrounded by aromatic residues (PHE188, PHE345, PHE346, HIS349, and TYR365). At the same time, the PCPMA motif formed a stable hydrophobic interaction by π-π stacking with PHE345 and PHE346. And the protonated nitrogen atom of the linker chain formed a salt bridge or hydrogen bonds with the carboxylate of ASP110, offering a critical strong interaction, which was manifested in **D1**, **D2**, **D3**, and **D4**. Furthermore, the right aryl substituent was oriented toward the secondary binding pocket (SBP), which was constructed by extracellular loop1, loop2, and helices I, II, and VII; the nitrogen atoms of compound **D3** in the amide bond of the linker formed hydrogen bonds. Moreover, in compound **D4**, hydrogen bonds and π-π stacking were formed between the nitrogen atom and the phenyl of the benzimidazole group with TYR365, which further stabilized the binding conformation, while the template compound **30r** and compound **D2** reversed the typical binding conformation at the OBS and SBP, which may be related to the biological activity of the compounds [17]. (The binding model of compound **30r** with D_3_R can be seen in Figure S1.) On the other hand, there were basic interactions of hydrogen bonds and salt bridges between the above two compounds and the residues, ensuring the affinity of the compounds with D_3_R (see Figure 7 and Figure S1). Overall, the docking poses of all compounds showed strong binding to D_3_R, highlighting the importance of specific interactions in the D_3_R–PCPMA ligands complex that drove their biological efficacy.

This suggests that the designed compounds have enhanced binding capabilities, allowing them to interact more effectively and stably with the protein. The notable improvement in binding interactions implies that these compounds may form stronger interactions within the active site, which could lead to improved biological efficacy. Further analysis of these interactions would provide insights into the specific binding modes and contribute to the rational design of more potent ligands.

### 2.6. Molecular Dynamics (MD) Simulation

**Figure 8:**
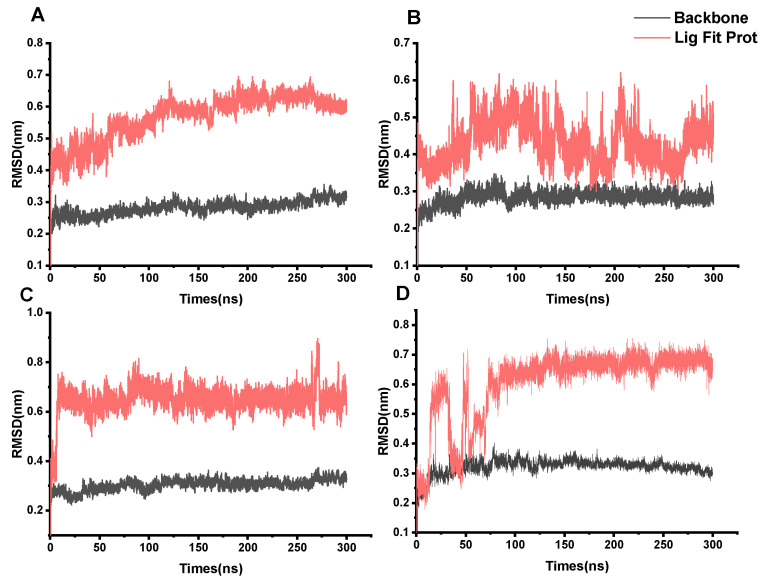
The RMSD of molecular dynamics simulations for the newly designed (**A**) compound **D1**, (**B**) compound **D2**, (**C**) compound **D3**, and (**D**) compound **D4**. Lig Fit Prot: Ligand-fitted protein.

To verify the binding modes under dynamic conditions, 300 ns MD simulations were conducted for the best docking pose of the template compound **30r** and the newly designed compounds **D1**, **D2**, **D3**, and **D4** with D_3_R. The root mean square deviation (RMSD), the radius of gyration (Rg), the root mean square fluctuation (RMSF), and the solvent-accessible surface area (SASA) of the MD results were also analyzed. As shown in Figure 8, all designed compounds reached a stable binding state with the protein complex system after 125 ns of simulation, and the RMSD values fluctuated steadily between 0.25 and 0.3 nm. The D_3_R-**D1** complex maintained a lower standard deviation (the RMSD for compound **D1** was 0.282 ± 0.022; for **D2**, 0.283 ± 0.0181; for **D3**, 0.303 ± 0.0225; and for **D4**, 0.321 ± 0.026). Compared with the template compound **30r** (see Figure S2), the newly designed compounds exhibited greater dynamic stability. Furthermore, during the whole simulation process, the RMSD of the D_3_R-**D1** complex system had the lowest fluctuation and was more stable, indicating that the ligand–protein binding ability was stronger and the composite system was more stable than the other three systems, which is consistent with the docking results. These findings collectively indicate that the newly designed compounds exhibited a high affinity for D_3_R, establishing them as promising candidates for further investigation in the development of drugs targeting this receptor.

The RMSF values for the four systems are shown in Figure S3A: all systems exhibited the same trend, with the RMSF fluctuation trend being lower than that of the template compound **30r**. The Rg characterizes the tightness of a protein structure and the change in the degree of peptide chain looseness of the protein during the simulation process. The results are shown in Figure S3B: with the exception of compound **D4**, the Rg fluctuation trends were more stable than those of the template compounds, suggesting that the four compounds bind to D_3_R with less overall change in the protein and less probability of folding and unfolding phenomena occurring. In addition, the SASA of the protein–ligand complexes were analyzed from the simulation trajectories to evaluate changes in the protein during simulation via each complex. As shown in Figure S3C, the SASA fluctuation trend was similar to that of the template compounds, except for compound **D4**, suggesting that the dynamic conformational changes of the protein expanded and contracted with the same trend throughout the simulations. Such characteristics were crucial for understanding the binding interactions and the potential functional implications of these compounds as therapeutic agents targeting D_3_R.

### 2.7. MMGB/PBSA Calculation of Binding Free Energy

To further measure the strength of interactions between the PCPMA ligands and D_3_R, MM/PBSA calculations were performed to explore the binding affinities for each ligand–D_3_R complex system. The last 25 ns of the MD simulation results were selected as the steady state to calculate the binding free energy, with the results shown in Table 4 Δ*G*_vdW_ and Δ*G*_polar(PB)_ contributed to attractive interactions, while ∆*G*_sol_ was repulsive to the total binding free energies.

The Δ*G*_tota_l values of compounds **D1** and **D2** were lower than that of the template compound **30r**, indicating more stable binding and stronger interaction with D_3_R. In contrast, the **D3** and **D4** systems had relatively high binding free energy, suggesting that these compounds may not bind as effectively as the template compound **30r** to the protein. Notably, it could be seen that Δ*G*_vdW_ was identified as important for ligand–receptor binding. In addition, Δ*G*_polar_ was detrimental to the binding affinity of the complexes because the large volume of the ligand binding pocket was exposed to a large amount of solvent.

The compound **30r** PCPMA backbone is more exposed to solvents, resulting in higher polar solvation energy. The nitrogen atom of the PCPMA in compound **D1** formed hydrogen bonds and salt bridges with ASP110, which enhanced its binding affinity with the proteins. This interaction led to a relatively smaller solvation energy and, therefore, a low total binding free energy. In compound **D2,** urea was added to the aliphatic linkage chain, enabling two nitrogen atoms to form hydrogen bonds and salt bridges with ASP110, and oxygen atoms formed hydrogen bonds with ILE183, which enhanced the binding interaction. At the same time, the molecule exhibited a strong electrostatic interaction, resulting in a larger Δ*G*_gas_ value. However, due to the PCPMA backbone being more exposed to solvents, a larger Δ*G*_polar(PB)_ was obtained, leading to a relatively higher binding free energy. Compound **D3** incorporated a large 2-(naphthale-2-yl)-ethyl group into the side chain of nitrogen atoms in the PCPMA framework, which allowed the molecule to better occupy the binding pocket. Furthermore, the introduction of triazole groups enabled the nitrogen atoms to form hydrogen bonds with ASN352, which enhanced the binding force, resulting in a higher Δ*G*_vdW_. In compound **D4**, the nitrogen atom of the imidazole group formed a hydrogen bond with TYR365, developing a strong electrostatic interaction, whereas a significant portion of the molecule was exposed to the solvent, leading to a larger Δ*G*_polar(PB)_.

**Figure 9:**
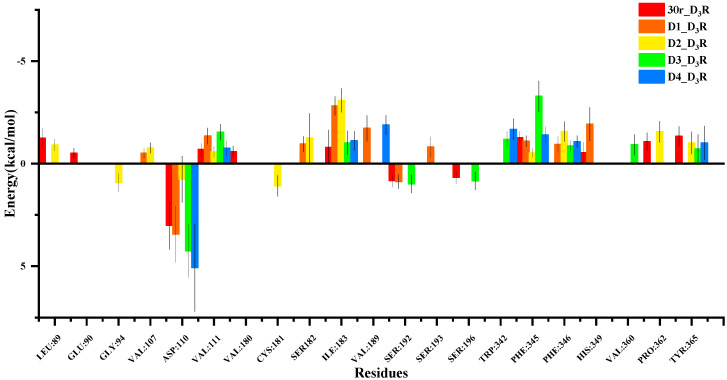
Binding free energy decomposition for the four protein–ligand complexes.

To further identify the key residues of the D_3_R–PCPMA ligand interactions, the binding energies obtained from the MM–PBSA analysis were further decomposed into the contributions of per residue. The key residues that contributed to the binding free energy for the D_3_R compounds **D1**, **D2**, **D3**, and **D4** complexes are shown in Figure 9. The analysis reveals that several residues in the vicinity of the binding site engaged in strong interactions with the designed compounds. Notably, the following residues were identified as having high energetic contributions to the binding free energy, thus playing critical roles in stabilizing the complexes: TYR26, LEU89, GLY94, VAL95, VAL98, VAL107, ASP110, VAL111, SER182, ILE183, PHE188, VAL189, SER192, SER196, PHE345, PHE346, HIS349, VAL360, PRO362, TYR365, SER366, THR369, and TYR373. According to the binding free energy decomposition results, the amino acid residues in the docking pocket contributed greatly to the binding free energy, and ASP110, which determines the affinity, was more prominent. These residues collectively demonstrated significant energetic contributions, indicating their involvement in various interactions that stabilize the ligand–receptor binding.

## 3. Materials and Methods

### 3.1. 3D-QSAR Analysis Dataset

A total of 50 different PCPMA derivatives were selected in this work, all of which were PCPMA derivatives reported by Cheng’s group [23]. Compound **30r**, with the best affinity, was selected as the template molecule, and Gaussian 09 was used to optimize the structure at the level of B3LYP/6-31G(d) [42,43]. The other 49 molecules were constructed by changing the moieties of **30r** and partially structurally optimized with Gaussian 09 (in which the common skeleton was fixed). All molecules were categorized into different classes, and one molecule at a time from each different type was selected for the test set. At the same time, the compounds were divided into a training set (40 compounds, 80%) and a test set (10 compounds, 20%), ensuring that both sets were structurally diverse with high, medium, and low activity. The distributions of the p*K*_i_ values of the training and test sets are shown in Table S1.

### 3.2. Compound Preparation and Restructuring

After constructing the 3D structures of the molecules, energy minimization was carried out using the Sybyl-X 2.0 (SYBYL-X 2.0, Tripos International, St. Louis, MO, USA) software package with the Tripos force field and Gasteiger–Huckel charges and 0.005 kcal/mol gradient convergence conditions using the Powell conjugate gradient method with 1000 iterations. A common backbone-based ligand stacking method was chosen for backbone stacking [44,45].

### 3.3. 3D-QSAR Models Production

Stacked training set molecules were placed in a 3D cubic lattice with a grid spacing of 2.0 Å to generate CoMFA and CoMSIA descriptor fields. The CoMFA fields were generated using sp3 carbon probe atoms with a charge of +1.0, and a van der Waals radius of 1.52 Å at each grid point, calculated using Tripos, hydrogen bonding, and indicator force fields, using the Lennard–Jones potential term to calculate the space field and a Coulomb term to calculate the electrostatic field. The cutoff values for both the spatial and electrostatic fields were set to 30.0 kcal/mol, and the rest of the parameters were according to the reference values. The CoMSIA fields (with the probe charge of +1.0, radius of 1 Å, hydrophobicity of +1.0, and acceptor of hydrogen bonds of +1.0) were calculated using the standard settings to obtain the steric, electrostatic, hydrophobic, hydrogen bond acceptor, and hydrogen bond donor fields. The internal predictive ability of the CoMFA and CoMSIA models was performed on the training set using partial least squares (PLS) analysis. Cross-validation correlation coefficients (q^2^) were determined by a leave-one-out (LOO) cross-validation procedure, and correlation coefficients (r^2^) were calculated by non-cross-validation methods. All CoMFA and CoMSIA studies were conducted using Sybyl-X 2.0 software.

### 3.4. Design of Novel PCPMA-Like D_3_R-Selective Ligands and Their Affinity Prediction with D_3_R

After constructing and validating the 3D-QSAR model, the relationship between molecular structure and binding affinity with D_3_R was illustrated based on the contour map information provided by the above model. Furthermore, a series of novel PCPMA-like D_3_R-selective ligands could be designed and their binding affinity activity with D_3_R could be predicted. The process was completed by Sybyl-X 2.0 and the newly designed ligands were treated as per Section 2.2.

### 3.5. Pharmacokinetic ADMET and Drug Similarity Prediction

The designed PCPMA derivatives were further investigated using ADMET prediction to assess the properties such as bioavailability, solubility, plasma protein binding, etc. Several pharmacokinetic parameters such as absorption, skin penetration, blood–brain barrier penetration, cytochrome P450 (CYP), toxicity, synthetic accessibility, and drug similarity prediction were determined using ADMETlab 3.0 [34] (https://admetlab3.scbdd.com/, accessed on 10 March 2025).

### 3.6. Molecular Docking

Molecular docking was performed using the Schrodinger 2021-2 software. Firstly, the 3D structures of the compounds were established. Then the 3D structures of these compounds were energy minimized by Gaussian 09. The crystal structure of the D_3_R target protein for docking was obtained from the PDB database (http://www.rcsb.org, accessed on 1 March 2023); the PDB ID is 3PBL [4]. The D_3_R structure was a dimer of the same structure, the intracellular structural domain was excised, and all remaining structural elements were identical to the crystal structure. Since the aim of our study was to investigate the effects of different ligands on the receptor, using the same variations in the receptor still did not bias the results, and the fact of the ligand binding to the transmembrane structural domains instead of the intracellular structural domains had no effect on the computation results, so the A chain of the subsequent D_3_R receptor was used for docking calculations. Afterward, the D_3_R protein structure was prepared by the Protein Preparation Wizard module in the Schrodinger 2021-2 software. The protein was assigned bond orders, had hydrogens added, was protonated, and had all crystallographic water molecules removed, and restrained minimization was applied. The compounds were structurally optimized using Gaussian 09 at the B3LYP/6-31G(d) level before docking and then were prepared using LigPrep with default settings. The docking grid was prepared with Glide [46], defining the binding site by crystal ligands and setting the ligand diameter midpoint box to 20 Å on all three axes. Finally, all of these ligands were docked into the calculated receptor grid using the XP scoring function and enhanced sampling [47]. The docking results were visualized and analyzed in Maestro, and the best scoring poses were selected for further dynamic simulation analysis.

### 3.7. Molecular Dynamics Simulations (MD)

The ligand–D_3_R complexes with the lowest absolute binding free energy in molecular docking were selected for molecular dynamics simulations using Gromacs 2023 [48], employing the frog hopping Newton integral algorithm for equilibrium kinetic integration. During the simulation, the intracellular structural domain of the D_3_R structure was excised and all remaining structural elements were identical to the crystal structure. Amber14sb_parmbsc1 force field parameters were used to generate protein topologies, and ligands were firstly calculated using Multiwfn [49] for RESP2(0.5) (http://sobereva.com/476, accessed on 1 May 2024) charges, and then the Sobtop (http://sobereva.com/soft/Sobtop, accessed on 1 May 2024) software was used to generate the GAFF force field topology. The TIP3P water model was used to add solvent to the protein–ligand system. Then, cubic boxes were built and replenished with Na^+^/Cl^−^ to balance the system. The energy of the complex system was optimized using the 50,000-step most rapid descent method (SD) combined with conjugate gradient (CG) methods. After the system energy optimization was completed, 100 ps limiting dynamics simulations (including isovolumetric NPT simulations) were performed for the composite system at one atmosphere and a temperature of 298.15 °C. Finally, a 300 ns molecular dynamics simulation was performed (the simulation was performed with a time step of 2 fs and the simulation trajectory was saved every 50 ps), and the trajectories were used to analyze the root mean square deviation (RMSD), root mean square fluctuation (RMSF), solvent accessible surface area (SASA), radius of gyration (Rg), etc.

### 3.8. Combined Free Energy MMGB/PBSA Calculations and Per-Residue Free Energy Decomposition Analysis

The molecular mechanics/Poisson–Boltzmann surface area (MM-PBSA) [50] calculations were preformed to estimate the ligand–protein binding free energy after MD simulations using the gmx_MMPBSA [51] module of the AmberTools 20 software. Based on the trajectory file of the MD simulation, the last 25 ns of the stable trajectory was selected for binding energy calculation, and 1 frame was selected for every 100 ps, for a total of 250 frames. The binding free energy calculations (Δ*G*_total_) focused on the molecular mechanical energy (Δ*E*_mm_) and solvation free energy (Δ*G*_sol_), and entropy calculations (TΔS) for the binding free energy calculations were taken from previously published studies [52,53]. The molecular mechanical energy was further decomposed into van der Waals energy (Δ*E*_vdW_), electrostatic energy (Δ*E*_ele_) and solvation free energy (Δ*G*_sol_), the last of which was decomposed into the contribution of polar solvation energy and non-polar solvation energy. In order to estimate the decomposition parameters for the van der Waals (Δ*G*_vdW_), electrostatic (Δ*G*_ele_), polar (Δ*G*_polar_), and nonpolar (Δ*G*_nonpolar_) energies, the contribution of amino acid residues interacting with the receptor to binding free energy was studied.

## 4. Conclusions

As a promising target for the treatment of psychiatric diseases, research on the dopamine D_3_R receptor has primarily focused on the development of ligands with high selectivity and high affinity. Low selectivity would bring a series of adverse side effects, and low affinity would lead to the reduction of ligand efficacy; therefore, an in-depth computational study was conducted on the selective dopamine D_3_R ligands. In this study, predictive 3D-QSAR models were developed using CoMFA and CoMSIA methods with 50 selective D_3_R ligands to identify and design novel PCPMA derivatives as D_3_R ligands. Both CoMFA and CoMSIA models yielded satisfactory predictive outcomes, with q^2^ of 0.607 and 0.643, r^2^ of 0.981 and 0.899, respectively, evaluated by a series of statistical parameters. The results of the comparison between the CoMFA and CoMSIA models show that CoMFA was superior to the CoMSIA model, indicating that the electrostatic and steric fields had an important impact on the affinity between D_3_R and PCPMA ligands. The contour plots provided further information to illustrate the structure–activity relationships: when the side chains of the nitrogen atoms in the connecting chain were replaced by naphthalene, it was predicted to have higher affinity. The affinity of the compound increased when the group attached after the amide bond was a hydrophilic, negatively charged triazole and imidazole group, a finding which will assist in the rational design of new PCPMA derivatives. Additionally, based on the 3D-QSAR modeling results, four novel D_3_R ligands were designed, and the prediction results show that their affinity with D_3_R was improved. To further validate the above predictions, molecular docking and 300 ns molecular dynamics simulations were conducted to evaluate the stability of the D_3_R–ligand complexes. The MD results show low RMSD and RMSF values, which indicate conformational stability throughout the simulation, and the above stability was in good agreement with the 3D-QSAR model and the molecular docking results. Furthermore, the binding free energies calculated by the MM-PBSA method further confirmed that the binding affinities of the designed compounds **D1** and **D2** to D_3_R were higher than that of the template molecule.

Overall, this work provides useful guidance for the rational design of selective D_3_R ligands and better explains the complex structure–activity relationships that exist between these selective D_3_R ligands, which can then be used to design novel, more potent, and more stable D_3_R ligands, and provides a theoretical basis for the discovery of candidate molecules with potential antipsychotic activity to treat patients with a variety of diseases, such as Parkinson’s disease or schizophrenia.

## Figures and Tables

**Table 1:** The statistical parameters of CoMFA and the best CoMSIA models.

PLS Statistic	CoMFA	CoMSIA
q^2^	0.607	0.643
r^2^	0.981	0.889
SEE	0.094	0.207
F	149.222	69.912
N	10	4
Steric	0.393	0.375
Electrostatic	0.607	-
Hydrophobic	-	0.725
SEP	0.5	0.51
RMSE	0.28	0.35

Note: q^2^: Non-cross-validated correlation coefficient, r^2^: Cross-validated correlation coefficient, SEE: Standard error of the estimate, F: F-test value, N: Optimum number of components, SEP: Standard error of prediction, RMSE: Root mean square error. When q^2^ > 0.5, r^2^ > 0.8, SEE ≈ 1, and F > 100, it indicates that the model is reliable.

**Table 2:** Designed ligands based on 3D-QSAR model and prediction results.

Compounds	Structures	p*K*_i_ (Pre.)	
		CoMFA	CoMSIA
**30r**	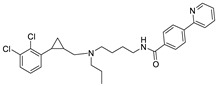	8.845	8.723
**D1**	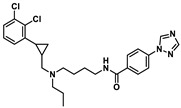	8.966	8.706
**D2**	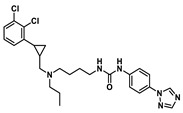	8.871	8.586
**D3**	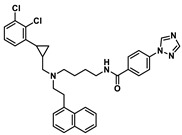	8.869	8.248
**D4**	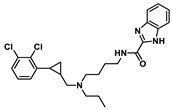	8.925	8.379

**Table 3:** Prediction of molecular property parameters of template molecule **30r** and the newly designed compounds.

	30r	D1	D2	D3	D4
Molecular weight	509.2	499.19	514.29	611.22	472.18
nRot	13	13	14	14	12
Flexibility	0.591	0.619	0.667	0.438	0.6
LogP	6.325	5.27	5.525	6.755	5.917
TPSA ^1^/Å^2^	45.23	63.05	75.08	63.05	61.02
SAscore ^2^	<6	<6	<6	<6	<6
nHA	4	4	7	6	5
nHD	1	1	2	1	2

nRot: number of rotatable bonds, optimal is 0–11; Flexibility: nRot/nRig, number of rotatable bonds/number of rings; ^1^ TPSA: Topological polar surface area, ^2^ SAscore: Synthetic accessibility score (SAscore < 6, easy to synthesize); nHA: number of hydrogen bond acceptors; nHD: number of hydrogen bond donors.

**Table 4:** The binding free energies of compounds **D1**, **D2**, **D3**, and **D4** to D_3_R and calculations for each energy term. All energies are expressed in kcal/mol.

System	30r	D1	D2	D3	D4
Δ*G*_vdW_	−72.88	−69.53	−62.28	−70.08	−58.04
Δ*G*_ele_	−16.68	−20.65	−46.98	−14.39	−37.35
Δ*G*_gas_	−89.56	−90.18	−109.26	−84.47	−95.39
Δ*G*_polar(PB)_	51.86	48.41	69.15	50.53	61.19
Δ*G*_nonpolar_	−6.28	−5.58	−6.27	−6.63	−5.56
Δ*G*_sol_	45.58	42.83	62.89	43.89	55.64
Δ*G*_total_	−43.98	−47.35	−46.36	−40.58	−39.75

## Data Availability

The data presented in this study are available in the article.

## Supplementary Materials

The following supporting information can be downloaded at: https://www.mdpi.com/article/10.3390/ijms26083559/s1. Refs. [54,55] are cited in Supplementary Materials.

## References

[1] Levant B. (1997). The D3 dopamine receptor: Neurobiology and potential clinical relevance. Pharmacol. Rev..

[2] Löber S., Hübner H., Tschammer N., Gmeiner P. (2011). Recent advances in the search for D3- and D4-selective drugs: Probes, models and candidates. Trends Pharmacol. Sci..

[3] Sokoloff P., Giros B., Martres M.-P., Bouthenet M.-L., Schwartz J.-C. (1990). Molecular cloning and characterization of a novel dopamine receptor (D3) as a target for neuroleptics. Nature.

[4] Chien E.Y.T., Liu W., Zhao Q., Katritch V., Han G.W., Hanson M.A., Shi L., Newman A.H., Javitch J.A., Cherezov V. (2010). Structure of the human dopamine D3 receptor in complex with a D2/D3 selective antagonist. Science.

[5] Sokoloff P., Giros B., Martres M.P., Andrieux M., Besancon R., Pilon C., Bouthenet M.L., Souil E., Schwartz J.C. (1992). Localization and function of the D3 dopamine receptor. Arzneimittelforschung.

[6] Zingales V., Torrisi S.A., Leggio G.M., Bucolo C., Drago F., Salomone S. (2021). Pharmacological and Genetic Evidence of Dopamine Receptor 3-Mediated Vasoconstriction in Isolated Mouse Aorta. Biomolecules.

[7] Reilly S.W., Riad A.A., Hsieh C.-J., Sahlholm K., Jacome D.A., Griffin S., Taylor M., Weng C.-C., Xu K., Kirschner N. (2019). Leveraging a Low-Affinity Diazaspiro Orthosteric Fragment to Reduce Dopamine D3 Receptor (D3R) Ligand Promiscuity across Highly Conserved Aminergic G-Protein-Coupled Receptors (GPCRs). J. Med. Chem..

[8] Keck T.M., John W.S., Czoty P.W., Nader M.A., Newman A.H. (2015). Identifying Medication Targets for Psychostimulant Addiction: Unraveling the Dopamine D3 Receptor Hypothesis. J. Med. Chem..

[9] Stemp G., Ashmeade T., Branch C.L., Hadley M.S., Hunter A.J., Johnson C.N., Nash D.J., Thewlis K.M., Vong A.K.K., Austin N.E. (2000). Design and synthesis of trans-N-4-2-(6-cyano-1,2,3,4-tetrahydroisoquinolin-2-yl) ethyl cyclohexyl -4-quinolinecarboxamide (SB-277011): A potent and selective dopamine D3 receptor antagonist with high oral bioavailability and CNS penetration in the rat. J. Med. Chem..

[10] Appel N.M., Li S.-H., Holmes T.H., Acri J.B. (2015). Dopamine D3 Receptor Antagonist (GSK598809) Potentiates the Hypertensive Effects of Cocaine in Conscious, Freely-Moving Dogs. J. Pharmacol. Exp. Ther..

[11] Nielsen S., Gowing L., Sabioni P., Le Foll B. (2019). Pharmacotherapies for cannabis dependence. Cochrane Database Syst. Rev..

[12] Gyertyán I., Kiss B., Sághy K., Laszy J., Szabó G., Szabados T., Gémesi L.I., Pásztor G., Zájer-Balázs M., Kapás M. (2011). Cariprazine (RGH-188), a potent D3/D2 dopamine receptor partial agonist, binds to dopamine D3 receptors in vivo and shows antipsychotic-like and procognitive effects in rodents. Neurochem. Int..

[13] Shonberg J., Lopez L., Scammells P.J., Christopoulos A., Capuano B., Lane J.R. (2014). Biased agonism at G protein-coupled receptors: The promise and the challenges—A medicinal chemistry perspective. Med. Res. Rev..

[14] Lane J.R., Sexton P.M., Christopoulos A. (2013). Bridging the gap: Bitopic ligands of G-protein-coupled receptors. Trends Pharmacol. Sci..

[15] Cao Y., Sun N., Zhang J., Liu Z., Tang Y.-Z., Wu Z., Kim K.-M., Cheon S.H. (2018). Design, synthesis, and evaluation of bitopic arylpiperazine-phthalimides as selective dopamine D3 receptor agonists. Medchemcomm.

[16] Shaik A.B., Boateng C.A., Battiti F.O., Bonifazi A., Cao J., Chen L., Chitsazi R., Ravi S., Lee K.H., Shi L. (2021). Structure Activity Relationships for a Series of Eticlopride-Based Dopamine D2/D3 Receptor Bitopic Ligands. J. Med. Chem..

[17] Arroyo-Urea S., Nazarova A.L., Carrión-Antolí Á., Bonifazi A., Battiti F.O., Lam J.H., Newman A.H., Katritch V., García-Nafría J. (2024). A bitopic agonist bound to the dopamine 3 receptor reveals a selectivity site. Nat. Commun..

[18] Dalton King H., Denhart D.J., Deskus J.A., Ditta J.L., Epperson J.R., Higgins M.A., Kung J.E., Marcin L.R., Sloan C.P., Mattson G.K. (2007). Conformationally restricted homotryptamines. Part 4: Heterocyclic and naphthyl analogs of a potent selective serotonin reuptake inhibitor. Bioorg. Med. Chem. Lett..

[19] Liu J., Clough S.J., Hutchinson A.J., Adamah-Biassi E.B., Popovska-Gorevski M., Dubocovich M.L. (2016). MT1 and MT2 Melatonin Receptors: A Therapeutic Perspective. Annu. Rev. Pharmacool. Toxicol..

[20] Cho S.J., Jensen N.H., Kurome T., Kadari S., Manzano M.L., Malberg J.E., Caldarone B., Roth B.L., Kozikowski A.P. (2009). Selective 5-Hydroxytryptamine 2C Receptor Agonists Derived from the Lead Compound Tranylcypromine: Identification of Drugs with Antidepressant-Like Action. J. Med. Chem..

[21] Carpenter J., Wang Y., Wu G., Feng J., Ye X.-Y., Morales C.L., Broekema M., Rossi K.A., Miller K.J., Murphy B.J. (2017). Utilization of an Active Site Mutant Receptor for the Identification of Potent and Selective Atypical 5-HT 2C Receptor Agonists. J. Med. Chem..

[22] Zhang G., Cheng J., McCorvy J.D., Lorello P.J., Caldarone B.J., Roth B.L., Kozikowski A.P. (2017). Discovery of N-Substituted (2-Phenylcyclopropyl)methylamines as Functionally Selective Serotonin 2C Receptor Agonists for Potential Use as Antipsychotic Medications. J. Med. Chem..

[23] Tan L., Zhou Q., Yan W., Sun J., Kozikowski A.P., Zhao S., Huang X.-P., Cheng J. (2020). Design and Synthesis of Bitopic 2-Phenylcyclopropylmethylamine (PCPMA) Derivatives as Selective Dopamine D3 Receptor Ligands. J. Med. Chem..

[24] Zhao L., Zhang L., Lei M. (2013). 3D-QSAR and docking studies on 2-arylbenzoxazole and linker-Y transthyretin amyloidogenesis inhibitors. Sci. China Chem..

[25] Damale M.G., Harke S.N., Kalam Khan F.A., Shinde D.B., Sangshetti J.N. (2014). Recent advances in multidimensional QSAR (4D-6D): A critical review. Mini Rev. Med. Chem..

[26] Lambrinidis G., Vallianatou T., Tsantili-Kakoulidou A. (2015). In vitro, in silico and integrated strategies for the estimation of plasma protein binding. A review. Adv. Drug Deliv. Rev..

[27] Kim K.H. (1995). Comparative molecular field analysis (CoMFA). Molecular Similarity in Drug Design.

[28] Klebe G., Abraham U., Mietzner T. (1994). Molecular similarity indices in a comparative analysis (CoMSIA) of drug molecules to correlate and predict their biological activity. J. Med. Chem..

[29] Völkner M., Wagner F., Steinheuer L.M., Carido M., Kurth T., Yazbeck A., Schor J., Wieneke S., Ebner L.J.A., Del Toro Runzer C. (2022). HBEGF-TNF induce a complex outer retinal pathology with photoreceptor cell extrusion in human organoids. Nat. Commun..

[30] Katritch V., Cherezov V., Stevens R.C. (2013). Structure-Function of the G Protein–Coupled Receptor Superfamily. Annu. Rev. Pharmacol. Toxicol..

[31] Chen L., Lin Y., Yan X., Ni H., Chen F., He F. (2023). 3D-QSAR studies on the structure–bitterness analysis of citrus flavonoids. Food Funct..

[32] Salas C.O., Zarate A.M., Kryštof V., Mella J., Faundez M., Brea J., Loza M.I., Brito I., Hendrychová D., Jorda R. (2019). Promising 2,6,9-Trisubstituted Purine Derivatives for Anticancer Compounds: Synthesis, 3D-QSAR, and Preliminary Biological Assays. Int. J. Mol. Sci..

[33] Zięba A., Laitinen T., Patel J.Z., Poso A., Kaczor A.A. (2021). Docking-Based 3D-QSAR Studies for 1,3,4-oxadiazol-2-one Derivatives as FAAH Inhibitors. Int. J. Mol. Sci..

[34] Fu L., Shi S., Yi J., Wang N., He Y., Wu Z., Peng J., Deng Y., Wang W., Wu C. (2024). ADMETlab 3.0: An updated comprehensive online ADMET prediction platform enhanced with broader coverage, improved performance, API functionality and decision support. Nucleic Acids Res..

[35] Smith D.A., Beaumont K., Maurer T.S., Di L. (2015). Volume of Distribution in Drug Design. J. Med. Chem..

[36] Hollenberg P.F. (1992). Mechanisms of cytochrome P450 and peroxidase-catalyzed xenobiotic metabolism. FASEB J..

[37] Lipinski C. (2012). Interview by Peter Kirkpatrick. Nat. Rev. Drug Discov..

[38] Ghose A.K., Viswanadhan V.N., Wendoloski J.J. (1999). A Knowledge-Based Approach in Designing Combinatorial or Medicinal Chemistry Libraries for Drug Discovery. 1. A Qualitative and Quantitative Characterization of Known Drug Databases. J. Comb. Chem..

[39] Plinski E.F., Plinska S. (2020). Veber’s Rules in Terahertz Light. https://www.researchsquare.com/article/rs-12857/v1.

[40] Egan W.J., Merz K.M., Baldwin J.J. (2000). Prediction of Drug Absorption Using Multivariate Statistics. J. Med. Chem..

[41] Muegge I. (2003). Selection criteria for drug-like compounds. Med. Res. Rev..

[42] Becke A.D. (1993). Density-functional thermochemistry. III. The role of exact exchange. J. Chem. Phys..

[43] Hariharan P.C., Pople J.A. (1973). The influence of polarization functions on molecular orbital hydrogenation energies. Theor. Chim. Acta.

[44] Lanka G., Begum D., Banerjee S., Adhikari N., P Y., Ghosh B. (2023). Pharmacophore-based virtual screening, 3D QSAR, Docking, ADMET, and MD simulation studies: An in silico perspective for the identification of new potential HDAC3 inhibitors. Comput. Biol. Med..

[45] Yan W., Lin G., Zhang R., Liang Z., Wu L., Wu W. (2020). Studies on molecular mechanism between ACE and inhibitory peptides in different bioactivities by 3D-QSAR and MD simulations. J. Mol. Liq..

[46] Repasky M.P., Shelley M., Friesner R.A. (2007). Flexible Ligand Docking with Glide. Curr. Protoc. Bioinform..

[47] Friesner R.A., Murphy R.B., Repasky M.P., Frye L.L., Greenwood J.R., Halgren T.A., Sanschagrin P.C., Mainz D.T. (2006). Extra Precision Glide: Docking and Scoring Incorporating a Model of Hydrophobic Enclosure for Protein-Ligand Complexes. J. Med. Chem..

[48] Van Der Spoel D., Lindahl E., Hess B., Groenhof G., Mark A.E., Berendsen H.J.C. (2005). GROMACS: Fast, flexible, and free. J. Comput. Chem..

[49] Lu T., Chen F. (2012). Multiwfn: A multifunctional wavefunction analyzer. J. Comput. Chem..

[50] Chen J., Zeng Q., Wang W., Sun H., Hu G. (2022). Decoding the Identification Mechanism of an SAM-III Riboswitch on Ligands through Multiple Independent Gaussian-Accelerated Molecular Dynamics Simulations. J. Chem. Inf. Model..

[51] Valdés-Tresanco M.S., Valdés-Tresanco M.E., Valiente P.A., Moreno E. (2021). gmx_MMPBSA: A New Tool to Perform End-State Free Energy Calculations w ith GROMACS. J. Chem. Theory Comput..

[52] Chohan T.A., Chen J.-J., Qian H.-Y., Pan Y.-L., Chen J.-Z. (2016). Molecular modeling studies to characterize N-phenylpyrimidin-2-amine selectivity for CDK2 and CDK4 through 3D-QSAR and molecular dynamics simulations. Mol. Biosyst..

[53] Chohan T.A., Qian H.-Y., Pan Y.-L., Chen J.-Z. (2016). Molecular simulation studies on the binding selectivity of 2-anilino-4 -(thiazol-5-yl)-pyrimidines in complexes with CDK2 and CDK7. Mol. Biosyst..

[54] Cramer R.D., Patterson D.E., Bunce J.D. (1988). Comparative molecular field analysis (CoMFA). 1. Effect of shape on binding of steroids to carrier proteins. J. Am. Chem. Soc..

[55] Kubinyi H. (1997). QSAR and 3D QSAR in drug design Part 1: Methodology. Drug Discovery Today.

