# Supplementary material for: 2-Phenylcyclopropylmethylamine (PCPMA) Derivatives as D3R-Selective Ligands for 3D-QSAR, Docking and Molecular Dynamics Simulation Studies
Source: Int J Mol Sci. 2025 Apr 10;26(8):3559. doi: 10.3390/ijms26083559 (PMC12026889; doi:10.3390/ijms26083559)
Supplement: Supplementary file 1 [file ijms-26-03559-s001.zip › ijms-3553636-supplementary.pdf]

Supplementary Materials

2-phenylcyclopropylmethylamines (PCPMAs) derivatives as D3R-selective ligands for 3D-QSAR, docking and molecular dynamics simulation studies

Li Guo[1], Yuepeng Gao[1], Sujuan Zhang[1], Lingmi Zhao[1], Runxin Zhao[1], Pinghua Sun[1, 3], Xinhui Pan[1,2]*, Wei Zhang[1]*

[1] School of Pharmacy/Key Laboratory of Xinjiang Phytomedicine Resource and Utilization Ministry of Education/Institute for Safflower Industry Research, Shihezi University, Shihezi, 832002, China.

[2] Stake Key Laboratory of Natural and Biomimetic Drugs, Department of Chemical Biology, School of Pharmaceutical Sciences, Peking University, Beijing, 100191, China.

[3] International Cooperative Laboratory of Traditional Chinese Medicine Modernization and Innovative Drug Development of Chinese Ministry of Education (MOE), College of Pharmacy, Jinan University, Guangzhou 510632, China.

*Email: zhangwei_1994@shzu.edu.cn (Wei Zhang)

Email: panshzu@shzu.edu.cn (Xinhui Pan)

Table of Contents

Figure S1. The best pose of template compound **30r** docking and corresponding 2D diagrams.

Figure S2. The RMSD of Molecular dynamics simulations for the template compound **30r.**

Figure S3. Molecular dynamics simulations of the four designed compounds and the template compound **30r** RMSF of the protein backbone atom Cα (A), Rg (B) and SASA (C) results.

Table S1. Structures, experimental and predicted binding affinities of 50 molecules in dataset.

Table S2. Possible results of CoMSIA Model.

Table S3. ADME/T properties of template and new compounds.

Table S4. Scoring results for the best conformations for molecular docking.

Brief Overview of 3D-QSAR Computational Methods and Statistical Terms

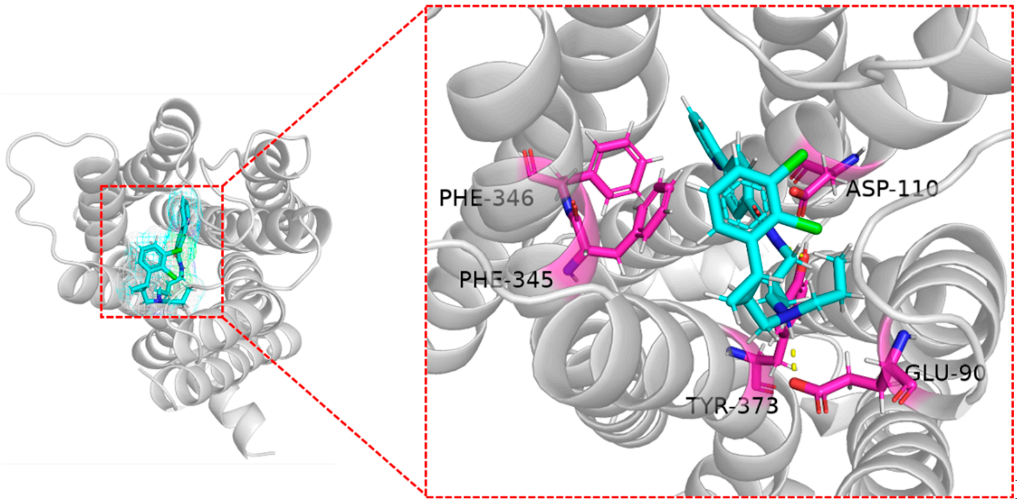

Figure S1. The best pose of template compound **30r** docking

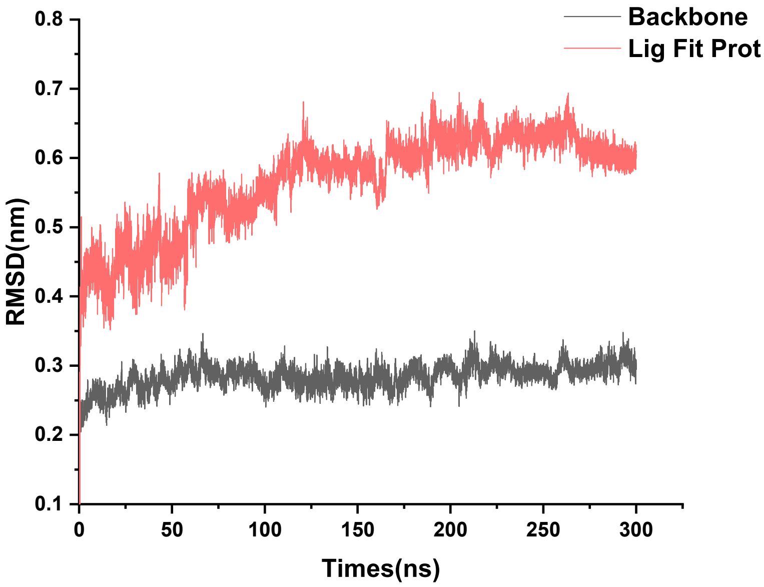

Figure S2. The RMSD of Molecular dynamics simulations for the template compounds **30r**.

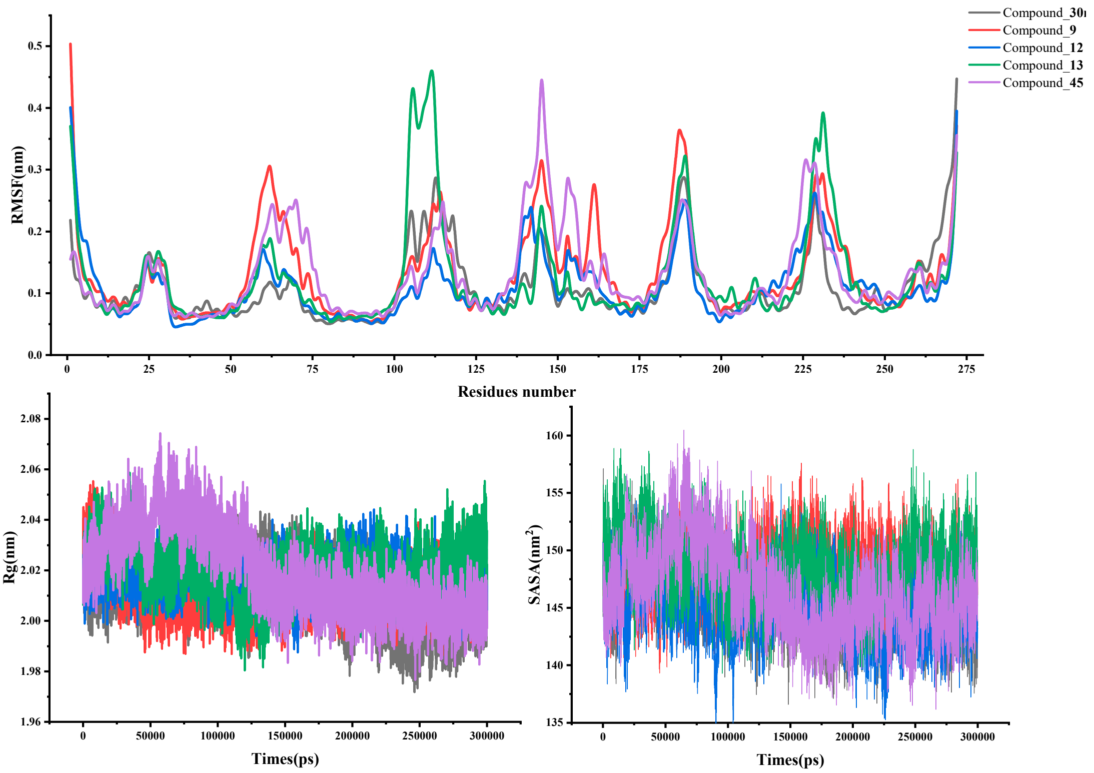

Figure S3. Molecular dynamics simulations of the four designed compounds and the template compound **30r** RMSF of the protein backbone atom Cα (A), Rg (B) and SASA (C) results.

Table S1. Structures, experimental and predicted binding affinities of 50 molecules in dataset.

Compounds[1]	Structures	p*K*i（Exp.）[1]	p*K*i（Pre.）		Sets
			CoMFA	CoMSIA	
**16a**	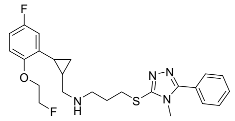	6.28	6.996	6.779	Test
**17a**	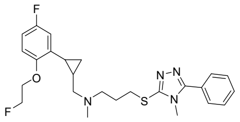	6.25	6.177	6.525	Training
**17b**	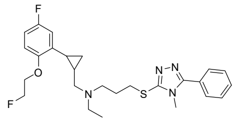	6.947	6.944	7.01	Training
**16b**	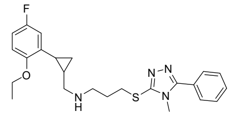	6.66	6.625	6.87	Training

Compounds[1]	Structures	p*K*i（Exp.）[1]	p*K*i（Pre.）		Sets
			CoMFA	CoMSIA	
**17c**	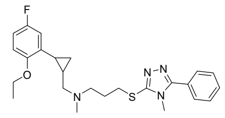	6.26	6.178	6.402	Training
**17d**	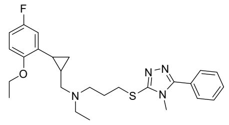	7.26	7.356	7.245	Test
**16c**	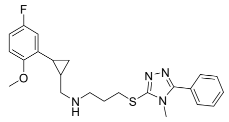	7.127	6.782	7.1	Training
**17e**	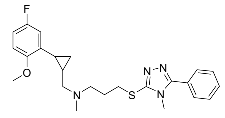	7.037	7.029	6.956	Training
**17f**	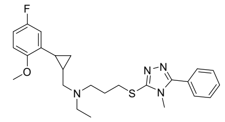	7.357	7.388	7.116	Training
**17g**	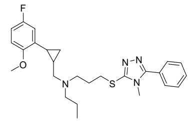	7.06	7.111	6.685	Training
**17h**	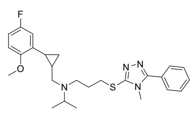	6.98	7.042	7.04	Training
**17i**	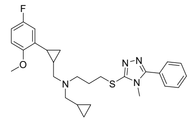	7.19	7.201	7.328	Training
**17j**	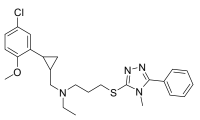	7.5	7.356	6.827	Test
**17k**	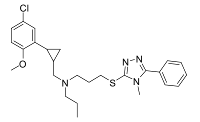	7.435	7.397	7.542	Training
**17l**	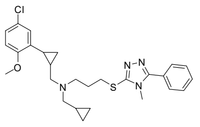	7.625	7.641	7.412	Training

Compounds[1]	Structures	pKi（Exp.）[1]	pKi（Pre.）		Sets
			CoMFA	CoMSIA	
**22a**	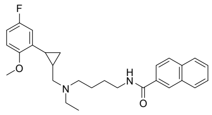	7.824	7.717	7.655	Training
**22b**	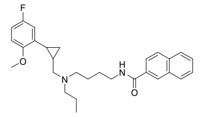	7.839	7.748	7.726	Training
**22c**	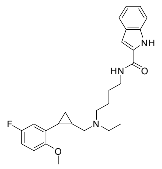	7.631	7.606	7.769	Training
**22d**	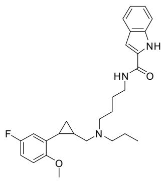	7.951	7.992	7.839	Training
**22e**	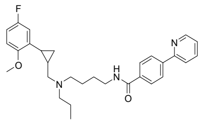	8.398	8.359	8.384	Training
**22f**	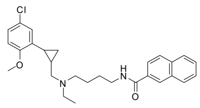	7.631	7.717	7.655	Training
**22g**	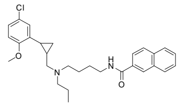	7.57	7.676	7.65	Training
**22h**	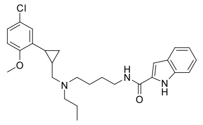	7.939	7.971	7.971	Training
**22i**	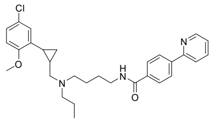	7.405	8.112	8.425	Test
**30a**	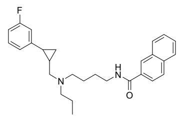	7.573	7.626	7.754	Training
**30b**	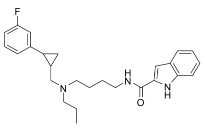	7.72	7.668	7.71	Training

Compounds[1]	Structures	p*K*i（Exp.）[1]	p*K*i（Pre.）		Sets
			CoMFA	CoMSIA	
**30c**	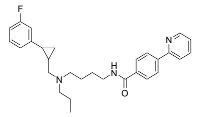	7.343	7.399	7.459	Training
**30d**	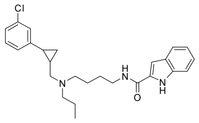	7.81	7.725	7.889	Training
**30e**	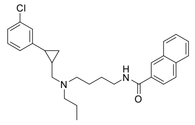	7.955	7.822	7.921	Test
**30f**	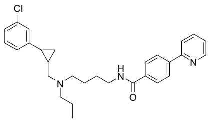	7.577	7.604	7.652	Training
**30g**	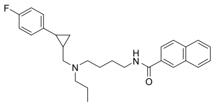	7.253	7.31	7.611	Training
**30h**	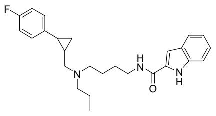	7.34	7.495	7.548	Training
**30i**	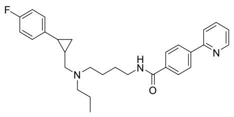	6.973	7.628	7.484	Test
**30j**	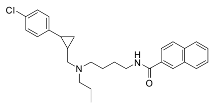	7.487	7.608	7.505	Training
**30k**	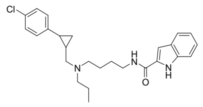	7.59	7.453	7.484	Training
**30l**	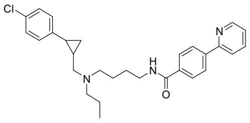	7.754	7.711	7.657	Training
**30m**	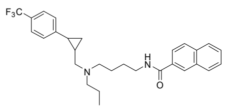	7.367	7.36	7.507	Training

Compounds[1]	Structures	p*K*i（Exp.）[1]	p*K*i（Pre.）		Sets
			CoMFA	CoMSIA	
**30n**	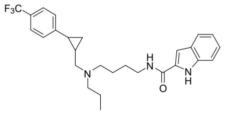	7.48	7.417	7.364	Training
**30o**	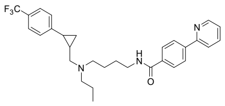	7.631	7.638	7.403	Training
**30p**	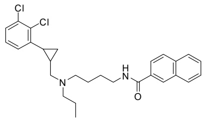	8.699	8.494	8.312	Training
**30q**	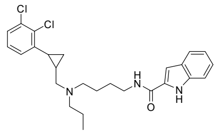	8.585	8.502	8.221	Training
**30r**	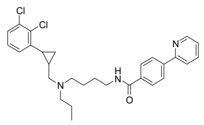	8.921	8.845	8.723	Training
(1*R*,2*R*)-**22e**	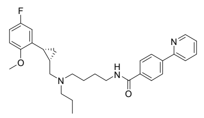	8.39	8.345	8.364	Training
(1*S*,2*S*)-**22e**	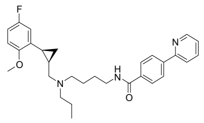	8.42	8.368	8.42	Test
(1*R*,2*R*)-**30p**	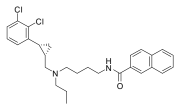	8.36	8.494	8.312	Training
(1*S*,2*S*)-**30p**	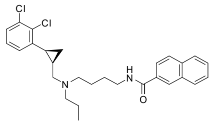	7.68	8.496	8.312	Test
(1*R*,2*R*)-**30q**	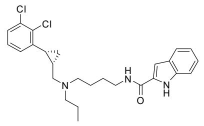	8.66	8.503	8.221	Test
(1*S*,2*S*)-**30q**	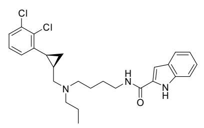	7.89	7.927	8.153	Training

Compounds[1]	Structures	p*K*i（Exp.）[1]	p*K*i（Pre.）		Sets
			CoMFA	CoMSIA	
(1*R*,2*R*)-**30r**	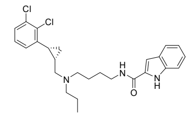	8.84	8.301	8.677	Test
(1*S*,2*S*)-**30r**	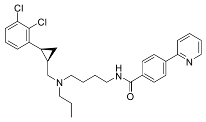	8.28	8.416	8.697	Training

Table S2. Possible results of CoMSIA Model.

Descriptors	q^2	r^2	N	F	SEE
D	0.428	0.660	5	13.208	0.368
A	0.524	0.783	7	16.528	0.303
H	0.639	0.907	5	66.424	0.192
S	0.467	0.798	4	34.634	0.279
E	0.552	0.744	2	53.833	0.306
EA	0.532	0.787	3	44.213	0.283
ED	0.52	0.755	3	36.936	0.303
EH	0.583	0.847	3	66.585	0.239
ES	0.535	0.760	2	58.535	0.296
SA	0.53	0.741	3	34.367	0.240
SD	0.542	0.853	8	22.438	0.253
**SH**	**0.643**	**0.889**	**4**	**69.912**	**0.207**
AH	0.618	0.779	2	65.112	0.226
AD	0.5	0.806	9	13.857	0.296
DH	0.604	0.903	6	51.406	0.199
SHE	0.587	0.850	3	67.968	0.237
SED	0.516	0.766	3	39.333	0.296
SEA	0.54	0.784	3	43.517	0.285

Descriptors	q^2	r^2	N	F	SEE
SHA	0.604	0.903	6	51.065	0.200
SHD	0.602	0.907	6	53.617	0.195
SAD	0.519	0.861	8	24.033	0.246
EAD	0.53	0.752	3	36.389	0.305
EAH	0.582	0.811	3	51.366	0.267
AHD	0.618	0.921	8	45.291	0.185
EDH	0.556	0.805	3	49.682	0.270
SEHD	0.559	0.820	3	54.759	0.260
SEHA	0.584	0.814	3	52.455	0.264
SHDA	0.612	0.931	8	52.175	0.174
EHDA	0.568	0.784	3	43.535	0.285
SEAD	0.53	0.756	3	37.147	0.303
SEHDA	0.569	0.789	3	44.966	0.281

Table S3 ADME/T properties of template and new compounds.

	Models	**30r**	**D1**	**D2**	**D3**	**D4**
	Caco-2 permeability	-5.222	-5.05	-5.272	-5.206	-5.103
	MDCK Permeability	-4.898	-4.827	-4.641	-4.819	-4.876
	PAMPA	0-0.1	0-0.1	0-0.1	0-0.1	0-0.1
	Pgp inhibitor	0.3-0.5	0.3-0.5	0.7-0.9	0.9-1.0	0.1-0.3
**A**	Pgp substrate	0-0.1	0-0.1	0-0.1	0.3-0.5	0-0.1
	HIA	0-0.1	0-0.1	0-0.1	0-0.1	0-0.1
	F20%	0-0.1	0-0.1	10-0.1	0-0.1	0-0.1
	F30%	0-0.1	0-0.1	0.5-0.7	0-0.1	0-0.1
	F50%	0.9-1.0	0.9-1.0	0.9-1.0	0.7-0.9	0.9-1.0
	PPB	96.0%	91.5%	90.3%	98.4%	95.6%
	VDss	0.98	0.792	0.612	0.469	0.803
	BBB	0.9-1.0	0.9-1.0	0.9-1.0	0.9-1.0	0.9-1.0
	Fu	3.0%	6.7%	6.5%	1.5%	3.8%
**D**	OATP1B1 inhibitor	0.9-1.0	0.7-0.9	0.9-1.0	0.3-0.5	0.7-0.9
	OATP1B3 inhibitor	0.9-1.0	0.9-1.0	0.9-1.0	0.7-0.9	0.9-1.0
	BCRP inhibitor	0-0.1	0-0.1	0-0.1	0-0.1	0.1-0.3
	MRP1 inhibitor	0.7-0.9	0.7-0.9	0.1-0.3	0.1-0.3	0.3-0.5

	Models	**30r**	**D1**	**D2**	**D3**	**D4**
**M**	BSEP inhibitor	0.9-1.0	0.9-1.0	0.7-0.9	0.9-1.0	0.9-1.0
	CYP1A2 inhibitor	0-0.1	0-0.1	0-0.1	0.7-0.9	0.1-0.3
	CYP1A2 substrate	0-0.1	0-0.1	0.7-0.9	0.9-1.0	0.9-1.0
	CYP2C19 inhibitor	0-0.1	0-0.1	0-0.1	0.1-0.3	0-0.1
	CYP2C19 substrate	0-0.1	0-0.1	0-0.1	0-0.1	0.1-0.3
	CYP2C9 inhibitor	0-0.1	0-0.1	0.1-0.3	0.9-1.0	0-0.1
	CYP2C9 substrate	0-0.1	0-0.1	0-0.1	0-0.1	0-0.1
	CYP2D6 inhibitor	0.9-1.0	0.9-1.0	0.9-1.0	0.9-1.0	0.9-1.0
	CYP2D6 substrate	0.9-1.0	0.9-1.0	0.9-1.0	0.9-1.0	0.9-1.0
	CYP3A4 inhibitor	0-0.1	0.9-1.0	0.9-1.0	0.9-1.0	0.7-0.9
	CYP3A4 substrate	0.9-1.0	0.9-1.0	0.9-1.0	0.9-1.0	0.9-1.0
	CYP2B6 inhibitor	0.9-1.0	0.9-1.0	0.9-1.0	0.9-1.0	0.3-0.5
	CYP2B6 substrate	0-0.1	0-0.1	0-0.1	0-0.1	0-0.1
	CYP2C8 inhibitor	0.9-1.0	0.9-1.0	0.9-1.0	0.9-1.0	0.9-1.0
	HLM Stability	0.1-0.3	0.9-1.0	0.9-1.0	0.9-1.0	0.9-1.0
**E**	CL_{plasma}	0.996	5.093	5.326	4.694	6.133
	T1/2	0.955	0.792	0.554	0.926	0.276
**T**	hERG Blockers	0.855	0.992	0.984	0.999	0.968
	hERG Blockers (10um)	0.666	0.945	0.912	0.984	0.932
	DILI	0.631	0.921	0.912	0.919	0.482
	AMES Toxicity	0.739	0.499	0.405	0.622	0.397
	Rat Oral Acute Toxicity	0.934	0.579	0.553	0.572	0.702
	FDAMDD	0.338	0.488	0.369	0.851	0.544
	Skin Sensitization	0.0	0.925	0.963	0.95	0.948
	Carcinogenicity	0.01	0.505	0.329	0.266	0.143
	Eye Corrosion	0.0	0.0	0.0	0.0	0.0
	Eye Irritation	0.01	0.003	0.003	0.0	0.024
	Respiratory	0.987	0.977	0.963	0.996	0.989
	Human Hepatotoxicity	0.912	0.869	0.815	0.916	0.722
	Drug-induced Nephrotoxicity	0.997	0.996	0.994	0.997	0.988
	Drug-induced Neurotoxicity	0.995	0.992	0.986	0.997	0.975
	Ototoxicity	0.938	0.96	0.952	0.981	0.916
	Hematotoxicity	0.418	0.436	0.511	0.443	0.414
	Genotoxicity	0.232	0.845	0.915	0.978	0.596
	RPMI-8226 Immunitoxicity	0.292	0.241	0.148	0.263	0.069
	A549 Cytotoxicity	0.767	0.551	0.293	0.708	0.423
	Hek293 Cytotoxicity	0.966	0.965	0.95	0.99	0.908
	BCF	1.503	1.442	1.488	1.507	1.426
	IGC50	4.286	3.921	3.845	4.222	4.068
	LC50DM	5.564	5.242	4.951	5.491	5.274

Models	**30r**	**D1**	**D2**	**D3**	**D4**
LC50FM	5.095	4.721	4.523	5.019	4.844

Note: 0-0.1、0.1-0.3-excellent，0.3-0.5、0.5-0.7-medium，0.7-0.9、0.9-1.0-poor.

Table S4 Scoring results for the best conformations for molecular docking.

	**D2**		**D3**	
	Docking score	XP GScore	Docking score	XP GScore
**30r**	-8.874	-8.874	-7.328	-7.328
**D1**	-7.929	-7.930	-8.683	-8.684
**D2**	-6.274	-6.275	-6.854	-6.855
**D3**	-8.154	-8.157	-9.994	-9.997
**D4**	-8.401	-8.407	-9.446	-9.452

Brief Overview of 3D-QSAR Computational Methods and Statistical Terms

1. Molecular Modeling Methods

Comparative Molecular Field Analysis (CoMFA), CoMFA correlates biological activity with 3D molecular steric (van der Waals) and electrostatic (Coulombic) fields generated by aligned molecules.

Workflow:

Align molecules to a common scaffold; Calculate steric/electrostatic fields using a probe atom; Derive a 3D-QSAR model via Partial Least Squares (PLS) regression; Output: Contour maps highlight regions where steric/electrostatic changes enhance or reduce activity[2].

Comparative Molecular Similarity Indices Analysis (CoMSIA), CoMFA by incorporating additional fields (hydrophobic, hydrogen-bond donor/acceptor) and using a Gaussian distance-dependent function for smoother sampling, Avoids singularities in steric/electrostatic potentials and provides more intuitive contour maps[3].

2. Statistical Terms

Correlation Coefficients

q^2 (LOO cross-validated r^2): Predicts model robustness via Leave-One-Out cross-validation. Values > 0.5 suggest predictive ability.

r^2 (conventional correlation coefficient): Measures goodness-of-fit for the training set. Values closer to 1 indicate stronger agreement.

Optimum Number of Components (N)

Determined during PLS regression to balance model complexity and overfitting. The ONC maximizes q^2 while minimizing standard error.

Standard Error of Estimate (SEE)

Quantifies precision of the model's predictions for the training set. Lower SEE indicates higher accuracy.

F: F-test value, reflects the significance of the linear relationship between model-predicted activity and measured activity $F > 10$: reliable model, $F > 100$: very strong statistical significance[4].

SEP: Standard Error of Prediction, SEP represents the average error of the model's prediction of the activity of the compounds in the test set, which directly reflects the prediction error of the model on the unknown compounds, $SEP < 0.5$: excellent prediction ability, $0.5 \leqslant SEP \leqslant 0.7$: acceptable range, $SEP > 0.7$: model needs to be optimized[4].

1. Tan, L.; Zhou, Q.; Yan, W.; Sun, J.; Kozikowski, A.P.; Zhao, S.; Huang, X.-P.; Cheng, J. Design and Synthesis of Bitopic 2-Phenylcyclopropylmethylamine (PCPMA) Derivatives as Selective Dopamine D3 Receptor Ligands. *J. Med. Chem.* **2020**, *63*, 4579-4602, doi:10.1021/acs.jmedchem.9b01835.
2. Cramer, R.D.; Patterson, D.E.; Bunce, J.D. Comparative molecular field analysis (CoMFA). 1. Effect of shape on binding of steroids to carrier proteins. *J. Am. Chem. Soc.* **1988**, *110*, 5959-5967, doi:10.1021/ja00226a005.
3. Klebe, G.; Abraham, U.; Mietzner, T. Molecular similarity indices in a comparative analysis (CoMSIA) of drug molecules to correlate and predict their biological activity. *J. Med. Chem.* **1994**, *37*, 4130-4146, doi:10.1021/jm00050a010.
4. Kubinyi, H. QSAR and 3D QSAR in drug design Part 1: methodology. *Drug Discovery Today* **1997**, *2*, 457-467, doi:10.1016/S1359-6446(97)01079-9.
